# Immune-mediated necrotizing myopathy (NAM) related to SARS-Cov-2 infection: a case report

**DOI:** 10.1186/s12883-023-03170-1

**Published:** 2023-03-22

**Authors:** Azliza Ibrahim, Wan Syamimee Wan Ghazali, Anna Misyail, Liyana Najwa, Abdul Hanif Khan, Wan Muhamad Amir, Alvin Oliver Payus, Loh Wei Chao, Janudin Baharin, Nor Shuhaila Shahril, Suryati Mohd Yusoff, Wan Aliaa, Hoo Fan Kee, Hamidon Basri

**Affiliations:** 1 Department of Neurology, Hospital Pengajar Universiti Putra Malaysia, Kuala Lumpur, Malaysia; 2 Department of Medicine, Faculty of Medicine and Health Sciences, Universiti Putra Malaysia, Kuala Lumpur, Malaysia; 3 Department of Neurology, Faculty of Medicine and Health Sciences, Universiti Putra Malaysia, Kuala Lumpur, Malaysia; 4 Department of Medicine, Hospital Pengajar Universiti Putra Malaysia, Kuala Lumpur, Malaysia; 5 Department of Medicine Based, Faculty of Health Sciences, Malaysia Sabah University, Sabah, Malaysia; 6 Department of Medicine, Hospital Putrajaya, Putrajaya, Malaysia; 7 Department of Pathology, Hospital Kuala Lumpur, Kuala Lumpur, Malaysia

**Keywords:** Necrotizing autoimmune myositis, Case report, COVID-19, Myalgia, Creatinine kinase

## Abstract

**Background:**

There is a growing body of evidence that severe acute respiratory syndrome coronavirus-2 (SARS-CoV-2) or COVID-19 infection is associated with the development of autoimmune diseases. A recent systematic review reported that the new-onset autoimmune disorders during or after COVID-19 infection included inflammatory myopathies such as immune-mediated necrotizing myopathies.

**Case presentation:**

We described a 60-year-old man diagnosed with COVID-19 infection and later presented with a two-week history of myalgia, progressive limb weakness, and dysphagia. He had a Creatinine Kinase (CK) level of more than 10,000 U/L, was strongly positive for anti-signal recognition particle (SRP) and anti-Ro52 antibody, and a muscle biopsy revealed a paucity-inflammation necrotizing myopathy with randomly distributed necrotic fibers, which was consistent with necrotizing autoimmune myositis (NAM). He responded well clinically and biochemically to intravenous immunoglobulin, steroids and immunosuppressant and he was able to resume to his baseline.

**Conclusion:**

SARS-CoV-2 may be associated with late-onset necrotizing myositis, mimicking autoimmune inflammatory myositis.

## Background

Myalgia is common in any viral illness including COVID-19 particularly during the prodromal phase which later resolves spontaneously. Infections are known to be a predisposing factor to immune dysregulation mainly via molecular mimicry, leading to autoantibody production and triggering autoimmune and autoinflammatory diseases. COVID-19-related myositis, also described as skeletal muscle injury and rhabdomyolysis, has been reported in up to 10% of infected patients [1]. In a case–control autopsy study, muscle biopsies from 26 of 43 individuals (60%) who had died with a diagnosis of COVID-19 demonstrated signs of muscle inflammation, ranging from mild to severe inflammatory myopathy [2]. The exact mechanism of myositis in COVID-19 is not well understood, but the exaggerated inflammatory response and the direct viral infection to the skeletal cells are the possible pathophysiology. There is very limited literature on autoimmune myositis triggered by COVID-19 infection. A systematic review highlighted nine cases of inflammatory myositis associated with COVID-19 infection, of which two were immune-mediated necrotizing myopathy [3]. Necrotizing autoimmune myopathy (NAM) is a rare subset of idiopathic inflammatory myopathies with a clinical presentation of severe proximal muscle weakness, elevated creatine kinase, myofiber necrosis with minimal inflammatory cell infiltrate on muscle biopsy, and infrequent extra-muscular involvement [4]. Its aetiology is unknown however, it has been linked to statin use, malignancy and autoantibodies such as anti-signal recognition particle (SRP) and anti-hydroxy-3-methylglutarul-CoA reductase (HMGCR) antibodies [5]. We report a patient diagnosed with necrotizing autoimmune myopathy who developed delayed onset progressive muscle weakness following a COVID-19 infection.

## Case presentation

A 60-year-old man presented with a two-week history of myalgia, progressive distal limb weakness, head drop, and dysphagia. He had no other significant symptoms. Past medical history revealed that four months prior, he was hospitalized for severe COVID-19 infection confirmed by a positive SARS-CoV-2 polymerase chain reaction (PCR) test, complicated by a superimposed bacterial infection which requiring a short stay in the intensive care unit for mechanical ventilation, corticosteroid and antibiotic therapy. He had no other medical illness and was not taking any medications. Clinical examination revealed symmetrical weakness of bilateral elbow, wrist, knee, and ankle movements with Medical Research Council (MRC) muscle power of grade 3/5 and extensor neck muscle weakness with no apparent muscle atrophy or fasciculation. The reflexes were reduced in both upper and lower extremities with down going plantar responses. His swallowing was impaired, and he required nasogastric tube feeding. The sensory, cranial nerve and other systemic examinations were normal. There was no autonomic dysfunction.

**Fig. 1:**
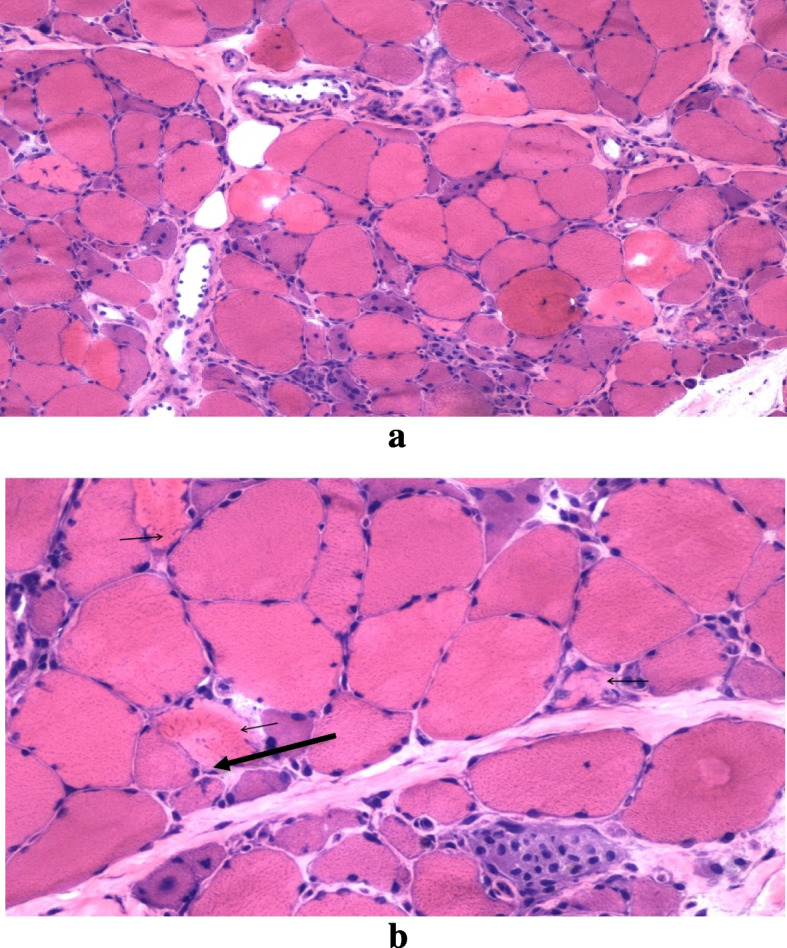
**a** Histological and immunohistochemical (H&E) analysis. H&E of the vastus lateralis muscle biopsy shows marked fiber size variation with many necrotizing and regenerating fibers. **b** Histological and immunohistochemical (H&E) analysis. H&E of the vastus lateralis muscle biopsy shows the necrotic fibres (arrow) are randomly distributed throughout the biopsy and has different stages of necrosis with paleness and coarse features

Routine blood investigations including complete blood count, renal profile, metabolic profile, infective markers, urinalysis, electrocardiogram (ECG), and echocardiogram were all normal. Chest X-ray showed opacities at bilateral upper lobes (left > right), and lower lobe. He had a raised Creatinine Kinase (CK) level of 13671 U/L and a positive anti-nuclear antibody (ANA) titre greater than 1:1280 with cytoplasmic dense fine speckled pattern. Rheumatoid Factor, complement levels, Hepatitis and HIV screening were all negative. Cerebrospinal fluid analysis of pathogens and autoimmune antibodies were also negative. Initially, the clinical myopathy was thought to be attributed to rhabdomyolysis; however, despite aggressive fluid hydration, the CK level remained consistently above 10,000 U/L. Electromyography (EMG) over bilateral deltoid and tibialis anterior muscles, revealed predominant fibrillation and positive sharp waves, consistent with electrophysiological evidence of myositis. Myositis-specific autoantibodies test panel were strongly positive for anti-signal recognition particle (SRP) and anti-Ro52 antibody. The muscle biopsy from the vastus lateralis demonstrates a pauci-inflammation necrotizing myopathy with randomly distributed necrotic fibres with no specific features, such as perifascicular atrophy, lymphocytic invasion in myofibers or rimmed vacuoles, to suggest dermatomyositis, polymyositis or inclusion body myositis, respectively (Fig. 1a and b). Computed Tomography (CT) scans of the thorax, abdomen, pelvis, and esophagogastroduodenoscopy (OGDS) were normal, excluding malignancy as a possible cause of immune-mediated necrotizing myopathies. However, the High-Resolution CT thorax revealed bilateral lower lobes reticulation with traction bronchiectasis suggestive of progressive lung fibrosis.

In view of the involvement bulbar and neck muscles, he was commenced on intravenous methylprednisolone 500 mg daily for 3 days and intravenous immunoglobulin 2 g/kg over five days followed by an immunosuppressant, Azathioprine 150 mg daily (2 mg/kg), and oral prednisolone 60 mg daily (1 mg/kg of prednisolone. On follow-up, he continued to improve clinically and biochemically. His steroids were tapered gradually, and he responded well to treatment. His CK levels significantly decreased, nasogastric tube removed, and he could ambulate independently and resumed to his baseline by 6 months of treatment.

## Discussion and conclusions

COVID-19 myopathy can range from an acute viral myositis to the rare occurrences of rhabdomyolysis, or a severe form of immune-mediated necrotizing myopathy. The manifestations of COVID-19 related myositis ranged from direct virus-induced muscle disease to triggered autoimmunity causing muscle inflammation. Myalgia is a common musculoskeletal manifestation of COVID-19 infection, presenting in nearly half of all COVID-19-infected patients [6]. Myalgia with elevated creatine kinase (CK) levels were found to be more pronounced in patients with critical illness who required intensive care support than in mildly affected individuals [1, 2, 7]. A large follow-up study of COVID-19 survivors has shown that 2% to 3% of patients experienced late onset fatigue and myalgia, even six months after SARS-CoV-2 infection [8]. Several case reports have been published on necrotizing autoimmune myositis (NAM) also known as immune-mediated necrotizing myopathy following COVID-19 infection. In our case, the patient had a severe COVID-19 infection that required intubation and admission to a critical care unit. The onset of myositis and elevated CK level developed later in the course of disease. Initially, he was treated for rhabdomyolysis, but his CK level significantly increased, and his clinical weakness persisted despite of aggressive fluid resuscitation. A more specific test for myositis was performed, including myositis-specific autoantibodies, which were strongly positive for SRP and anti-Ro52 antibody, and a muscle biopsy, which was consistent with necrotising autoimmune myositis (NAM).

NAM is a rare subgroup of autoimmune inflammatory myositis and manifest acutely over days or weeks, or subacutely over months, causing severe weakness and extremely elevated creatine kinase (CK) levels in the thousands [9]. Histologically, NAM is distinguished from other inflammatory myositis by the presence of necrotic muscle fibers and a lack of inflammation [10]. Autoantibodies including Anti-Jo-1, anti-SRP and anti-HMGCR are associated with triggering NAM [11]. To the best of our knowledge, there were two published cases on covid-19 associated with NAM. The first case was an 88-year-old man who presented with acute bilateral thigh weakness and an elevated CK level of 13,581 U/L [8]. He was found to be positive for COVID-19, and his weakness and CK level improved weeks after being treated with hydroxychloroquine. Another 60-year-old man had a delayed onset of painful muscle weakness 1 week after acute covid-19 infection, which was associated with a very high CK of 11,842 U/L. He was subsequently improved clinically and biochemically with a course of intravenous immunoglobulin (IVIG) [8]. Both reported cases are highly suggestive of an autoimmune inflammatory myopathy within the NAM spectrum triggered by the virus. Referring to our patient, he has positive anti-Ro52, and his initial High-Resolution CT thorax (HRCT) showed some fibrotic lungs changes which suggested the possibility of Idiopathic Inflammatory Myositis. However, repeated HRCT two months after the Covid 19 showed resolution of the fibrotic changes which explained more of resolved organizing pneumonia and the presence of NAM on muscle biopsy with positive anti-Ro52 in this patient may directly associated with Covid 19.

The pathophysiology of myositis caused by viruses is not well understood. Muscle injury can occur following viral infection as a result of the complement system being activated by the deposition of virus–antibody complexes and circulating viral toxins [12]. A recent case–control autopsy study on patients who died from COVID-19 infection with a positive SARS-CoV-2 polymerase chain reaction or from other critical illnesses found that a majority of individuals with severe COVID-19 had mild to severe myositis, with skeletal muscle inflammation being more pronounced than cardiac inflammation [2]. Furthermore, the viral load detection was low or negative in most skeletal and cardiac muscles, suggesting that myositis is most likely caused by circulating viral ribonucleic acid (RNA) rather than genuine myocyte infection [2]. This suggests that SARS-CoV-2 may cause an immune-mediated or postinfectious myopathy. Interestingly, the angiotensin-converting enzyme 2 (ACE 2) receptors, which is the entry site of SARS-CoV-2, are present on skeletal muscles which necessitate further investigation to ascertain whether SARS-CoV-2 could be the first virus that can potentially cause direct muscle injury [8].

In conclusion, we present a rare case of inflammatory necrotizing myositis associated with SARS-CoV-2 IgG, demonstrating that prompt diagnosis and treatment significantly improved the patient's outcome.

## Data Availability

The figure images used in this case report are available from the corresponding author on reasonable request.

## Abbreviations

NAM: Immune-Mediated Necrotizing Myopathy
SARS-CoV-2: Severe acute respiratory syndrome coronavirus-2
CK: Creatinine Kinase
SRP: Anti-signal recognition particle
HMGCR: Anti-hydroxy-3-methylglutarul-CoA reductase antibodies
PCR: Polymerase chain reaction
MRC: Medical Research Council
ECG: Electrocardiogram (ECG)
ANA: Anti-nuclear antibody
EMG: Electromyography
CT: Computed Tomography
OGDS: Esophagogastroduodenoscopy
HRCT: High-Resolution CT thorax
ACE 2: Angiotensin-converting enzyme 2
RNA: Ribonucleic acid
